# *Ex vivo* tumor culture systems for functional drug testing and therapy response prediction

**DOI:** 10.4155/fsoa-2017-0003

**Published:** 2017-03-27

**Authors:** Titia G Meijer, Kishan AT Naipal, Agnes Jager, Dik C van Gent

**Affiliations:** 1 Department of Molecular Genetics, Erasmus Medical Center, Rotterdam, The Netherlands; 2 Department of Medical Oncology, Erasmus Medical Center, Rotterdam, The Netherlands

**Keywords:** cancer-on-chip, cell culture, functional drug testing, organoids, organotypic tissue slices, PDX, personalized medicine

## Abstract

Optimal patient stratification is of utmost importance in the era of personalized medicine. Prediction of individual treatment responses by functional *ex vivo* assays requires model systems derived from viable tumor samples, which should closely resemble *in vivo* tumor characteristics and microenvironment. This review discusses a broad spectrum of model systems, ranging from classic 2D monolayer culture techniques to more experimental ‘cancer-on-chip’ procedures. We mainly focus on organotypic tumor slices that take tumor heterogeneity and tumor–stromal interactions into account. These 3D model systems can be exploited for patient selection as well as for fundamental research. Selection of the right model system for each specific research endeavor is crucial and requires careful balancing of the pros and cons of each technology.

## Lay abstract

Selection of the right therapy for individual cancer patients is very important with the expanding number of possible treatments. How tumors respond to a therapy can be tested by treating a sample from the tumor outside the body. Various culture methods can be used to maintain this tumor sample. Each of these model systems has its own benefits and disadvantages. In this review, we discuss the advantages and drawbacks of the available model systems and how they can be used to guide personalized medicine.

Treatment of epithelial cancers generally comprises surgical resection, radiation and/or systemic therapy. Systemic therapies traditionally consist of chemotherapeutic agents. Recently, more and more targeted therapies, such as small molecule inhibitors and monoclonal antibodies, have been developed. Targeted therapies have the potential advantage that they are directed against specific characteristics unique to the tumor cells, leaving the surrounding healthy tissue relatively unharmed. Over the last decades, cancer treatment has moved from ‘one-size-fits-all’ regimens toward more personalized cancer therapy. Molecular characteristics of the tumor cells are now used for therapy selection. For example, the monoclonal antibody trastuzumab, targeting the human epidermal growth factor receptor 2 (HER2), dramatically improved survival for patients with breast tumors overexpressing HER2 [1]. These positive developments pose new challenges: proper selection of patients that are most likely to benefit from these targeted treatment regimens.

Adequate patient selection requires extensive molecular characterization of individual tumors. The search for predictive biomarkers started with specific molecular markers (e.g., *EGFR* mutation status in non-small-cell lung cancer [2]) and developed over time into genomic, transcriptional and proteomic signatures [3–5]. In the future, next-generation sequencing techniques will be exploited to characterize individual patients molecularly and predict therapy response. However, validation of these biomarkers and subsequent implementation in the clinic are major bottle-necks that require extensive research.

As therapy response often cannot be predicted accurately by a single genetic marker only, alternative ways of patient stratification are needed. Beyond mutational status, many other factors influence tumor behavior and therapy response, for example epigenetic factors and the tumor microenvironment [6,7]. For instance, although *HER2* amplification is a strong predictive marker of response to trastuzumab in breast cancer patients, its predictive value in gastric cancer is much weaker [8]. Therefore, the current difficulty to translate genetic information to tumor behavior necessitates development of tools to select patients for therapies based on tumor phenotype rather than genotype. *Ex vivo* assays that predict therapy response may fill this knowledge gap.

These functional assays require a viable sample from the tumor, which is then cultivated in the laboratory and exploited for drug screening or other *ex vivo* functional testing. Obviously, these tests require optimal model systems, which most closely resemble the *in vivo* tumor characteristics and microenvironment. Established tumor cell lines and genetically engineered mouse models are time consuming and do not represent the variation and heterogeneity observed in cancers from patients. Therefore, these models are usually not the optimal choice for development of assays to select patients for personalized cancer treatments [9]. Many alternative model systems are emerging to overcome these drawbacks and resemble *in vivo* tumors more closely. These model systems enable execution of various *ex vivo* functional tests that aim to predict therapy response in the patient. We here discuss generation of 2D and 3D tumor cell culture methods, patient-derived xenografts (PDX) and organotypic tumor tissue slices (Figure 1). We here review the benefits and disadvantages of the available (preclinical) cancer model systems.

**Figure 1.**
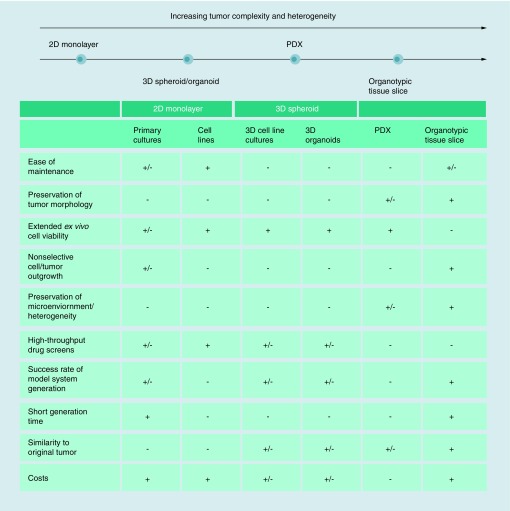
**Comparison of *ex vivo* tumor culture techniques.** Fresh viable tumor tissue can be preserved and cultured *ex vivo* in several ways, each having its own advantages and disadvantages. A tumor sample can be dissociated using enzymatic and/or mechanical methods and subsequently cultured either as a 2D monolayer or in a 3D tumor spheroid culture. To mimic the *in vivo* situation as much as possible, dissected tumor samples can be implanted in immunodeficient mice to generate patient-derived xenograft models. Organotypic tumor tissue slices can be generated by precision slicing of a tumor specimen, keeping general tumor/tissue architecture intact. +: Advantages of the method; -: Disadvantages of the method.

## 2D monolayer culture of dissociated tumor cells

To obtain a 2D monolayer of cells, the tumor is dissociated by specific proteolytic enzymes such as collagenase, dispase and/or trypsin. Depending on the tumor type, enzymatic digestion is combined with mechanical dissociation for better dispersal of the tumor mass [10]. Not all tumors can be cultured *ex vivo* in monolayers. The need to adhere to the culture dish obviously causes a selection bias for adherent cells. Two types of 2D monolayer cultures exist: primary (tumor) cell cultures and cancer cell lines. Primary cell cultures are heterogeneous and represent the original tumor more closely but do not possess the limitless proliferative capacity that cancer cell lines have. Cancer cell lines are defined as clonal outgrowths from a primary tumor cell culture.

Once dissociated tumor cells successfully form a 2D monolayer *in vitro*, characterization of these cells can be performed in various ways. Primary (tumor) cell cultures can be exploited for diagnostic testing. Compared with cancer cell lines, primary cell cultures have less clonal selection and allow several short-term functional analyses. This works well for some tumor types, such as bladder tumor cell cultures that have been used to characterize Nucleotide Excision Repair activity [10].

Primary (tumor) cell cultures can also be established from tumor cells found in body fluids, including ascites and pulmonary effusion. For example, withdrawal of excessive ascites from ovarian cancer patients is often performed regularly for symptom relief and, therefore, less invasive than tumor biopsies. Generation of 2D monolayer cultures from these tumor cells has a 90% success rate, thereby providing a model system for functional testing and guiding personalized medicine for these patients [11,12].

Human cancer cell lines have proved invaluable in both fundamental and translational research. Easy handling, homogeneous character and limitless growth make this the model system of choice for many large high-throughput experiments. High-throughput drug screenings using large panels of cancer cell lines have led to the discovery of new drug targets and gene signatures predicting therapy responses [13,14].

Successful establishment of cancer cell lines from solid tumors is often inefficient, because of failure to adhere to the culture dish or loss of proliferative capacity after a few passages (e.g., for breast cancer the success rate is between 1 and 10% [15]). Especially, slow growing tumors are severely underrepresented, as they do not often give rise to tumor cell lines. The optimal result is a clonal outgrowth and, therefore, cell lines do not represent the heterogeneity of the primary tumor. Indeed, cell lines and the *in vivo* tumors from which they originate, show many genetic, epigenetic and gene expression differences [16].

Another limitation of cell lines is the extended time required for clonal outgrowth, minimizing the applicability of this model as a patient selection tool for personalized medicine. Genetic drift and cross-contamination are other issues often encountered when working with cell lines. This is not a problem when using primary (tumor) cell cultures in low passages for diagnostic testing, but is a major concern for extended culturing of cell lines in a laboratory setting. The latter problem can be minimized by freezing representative low passage stocks [17].

In conclusion, 2D culture systems do not capture the subtleties of the original tumor microenvironment. However, primary tumor cell cultures may represent a valid approach to guide personalized medicine decision-making. Cancer cell lines are valuable tools for high-throughput drug screening, although translation of these screens to the clinic can be difficult.

## 3D tumor cell models

The limited cell–cell interactions in 2D monolayer cultures introduce major changes in cellular physiology. Therefore, 3D cultures of the same cells may represent the original organ or tumor more faithfully than traditional cell cultures. 3D cancer cell line-based models have been reviewed elsewhere [18]. Although they capture some features of tumor cell biology better than 2D culture systems, they fail to mimic tumor heterogeneity. For this reason, it would be preferred to start 3D cultures from primary tumor cells and/or tumor stem cells instead of cancer cell lines.

Some decades ago, collagen gels floating in the culture medium were shown to allow epithelial cells from different origins to form alveolus-like structures and maintain tissue function and differentiation [19]. This was the beginning of *ex vivo* culturing of normal epithelial cells, such as mammary acini and colonic crypts, as functional units.

More recently, these 3D culture systems have been adapted such that they can grow for many passages. Such organoids can be established through isolation of adult stem cells and subsequent embedding of the cells in a 3D matrix. The undifferentiated stem cells (e.g., Lgr5+ cells) are stimulated by supplements of tissue-specific exogenous growth factors, in addition to growth factors endogenously produced by the stem cell microenvironment and surrounding mesenchyme [20]. They self-organize into epithelia of the respective organ of origin, such as intestinal stem cells giving rise to formation of mini-guts, representing the epithelial architecture of the small intestine and colon [21].

Similar technology allows 3D culture of tumor cells in spheroid structures – often referred to as tumor organoids. This technique can achieve long-term *ex vivo* expansion of tumor cells that still represent the heterogeneity of the original tumor [22]. Tumor organoid growth can have a high success rate, even when starting material is limited [23]. Up to date, successful human tumor derived organoids have been created from many different tumor types, including colorectal, stomach, liver and pancreas cancers [22,24–26]. Recently, tumor organoids have also been grown from frozen material, greatly extending the applicability of this technique [27]. However, it remains to be demonstrated whether tumor organoids can be grown with similar efficiencies from other tumor types.

The introduction of organoid cultures has created novel opportunities for high-throughput drug screens aiding personalized cancer treatment, biomarker discovery and studies on drug resistance mechanisms. A living organoid biobank for colorectal cancer patients is currently being collected, allowing gene expression analysis to detect gene–drug associations. Ideally, drug screens on these tumor organoids point toward effective personalized treatment strategies [28].

However, some drawbacks of the technique have surfaced as well. The requirement of a collagen gel for 3D culturing was the initial break-through, yet seems to complicate potential drug screening and makes culturing more labor intensive.

Moreover, tumor organoids derived from a homogenous population of stem cells do not harbor the microenvironment of *in vivo* tumors, which also include nontransformed cells such as stromal fibroblasts and infiltrating immune cells. However, this technique can be developed further by introducing additional heterogeneity through patient-matched co-cultures with organoids grown from normal tissue adjacent to the tumor. Hybrid organoids consisting of tumor cells and stromal cells show promising potential for unraveling metastatic processes and tumor-stroma characteristics [29]. These co-cultures can also be adapted for other 3D culturing techniques to mimic the tumor microenvironment. For example, the development of 3D tumor co-cultures from cancer cell lines grown in combination with fibroblasts, endothelial cells, immune cells or bone cells enable cross-talk between tumor cells and the stromal cells of the microenvironment [30–33].

Organoid culture systems are suboptimal as a diagnostic tool, since their generation takes several weeks and clinical diagnostic testing for individual therapy selection should be conclusive within a much shorter time frame [28]. On the other hand, one could envision organoid generation from primary tumor or metastasis material of patients treated with chemotherapy. Simultaneous treatment of the tumor organoid with various therapeutics could guide further therapy selection for these patients. The correlation between organoid and *in vivo* tumor therapy response would require extensive validation in this case.

In conclusion, 3D organoid cultures are valuable tools for drug screens, biomarker discovery and studies on drug resistance mechanisms. Nevertheless, this model lacks the complexity of the tumor microenvironment and is less suited to guide personalized medicine.

## Patient-derived xenografts

Dissociation of the tumor tissue is a prerequisite for 2D monolayer cultures and tumor organoids. This leads to loss of tumor heterogeneity and outgrowth of a specific subset of tumor cells. Another method to expand and preserve individual tumors from cancer patients is implantation of fresh pieces of the tumor in immune-deficient mice, subcutaneously or in a place that more closely resembles the original tumor location [34,35]. PDX tumor models retain intratumor heterogeneity [36]. The first PDX models were generated in the 1980s and they are still important and widely used in cancer research [37]. PDX models have been exploited for drug screening, biomarker discovery, identification of resistance mechanisms and preclinical evaluation of (personalized) treatment strategies [34]. PDX models maintain several characteristics of the *in vivo* tumor, including histopathological features, gene expression profiles, copy number variation and metastatic behavior [38–41].

Systematic analysis of PDX models enables biobanking of genomically well-defined tumors [34]. These biobanks are valuable resources for developing new predictive or prognostic biomarkers and individualized treatment strategies, thereby potentially guiding personalized medicine [42]. Also, co-clinical trials have been designed, in which PDX models are treated with anticancer therapies in parallel with the same treatment of patients in clinical trials [43,44]. The co-clinical trial concept allows integration of preclinical and clinical data, facilitating personalized treatment selection for patients, discovery of predictive biomarkers and identification of resistance mechanisms. Whether responses to chemotherapy observed in PDX models resemble the response rates of patients in clinical trials still remain to be elucidated [45,46].

More recently, a pilot study with a similar concept was carried out. Treatments for patients with advanced cancer were selected on the basis of activity against a personalized tumorgraft derived from the *in vivo* tumor [47]. These personalized tumor graft models led to selection of a treatment regimen for 12 out of 14 patients. The treatments selected for each individual patient were not obvious and would not have been the first choice for a conventional second- or third-line treatment. In 9 out of 12 patients the selected treatment resulted in durable partial remission [47]. These results are quite striking, since the expected response rate with Phase I agents, the only available option for some of these patients, is less than 10% [48]. These results need to be confirmed in larger cohorts of patients to get a better idea of the level of concordance between response in personalized tumorgraft models and the tumor of origin.

While ingenious advancements have been made in PDX applications, PDX models still harbor some important disadvantages. The first major drawback is the variable success rate of tumor engraftment [47]. Therefore, the variation observed in the cancer patient population may not be recapitulated faithfully in PDX models due to this selective engraftment rate [34]. Clinically aggressive tumors with many proliferative cancer cells, have the highest engraftment rate [49,50].

A second major drawback is the long generation time of PDX models, which limits their use in personalized medicine. The time between implantation and progressive growth of the xenograft tumor (PDX generation time or tumor graft latency) can range from 2 to 12 months [51,52]. In case of metastasized disease, patients may not even survive the PDX generation time [51]. PDX models may have limited use in diagnostics due to their low-throughput character and relatively high costs.

In addition to these practical problems for use of PDX models in personalized medicine, their use is also somewhat limited because of fundamental imperfections of the model. Although they retain intratumor heterogeneity, they fail to maintain the heterogeneity in the human tumor microenvironment, as the tumor stroma is slowly substituted by mouse stroma upon passaging. Therefore, the contribution of tumor–stroma interaction cannot be deduced faithfully from PDX models for drug screening.

Furthermore, PDX formation requires tumor implantation in severely immunocompromised host animals, complicating the evaluation of tumor immunology and drugs targeting the immune system [53]. This problem could be circumvented by using mice carrying a humanized immune system, although problems with graft-versus-host disease limit this approach severely [54]. Thus, when studying immunotherapies or tumor–stromal interactions there is a need for alternative model systems that allow exploration of the tumor microenvironment.

Overall, PDX models harbor more intratumor complexity than 2D monolayers or various 3D culturing techniques because the tumor is not dissociated. Since the generation time of PDX models is rather long, this model is less suitable for drug screening and personalized medicine but is still important for drug validation, investigation of therapy resistance mechanisms and biomarker development.

## Organotypic tumor tissue slices

Various 3D culture systems have been designed to resemble *in vivo* tumors as closely as possible, taking tumor heterogeneity and tumor–stromal interactions into account. Most of these 3D culture approaches mimic tumor complexity only partially. The initial step for all techniques is dissociation of tumor tissue before the cells are stimulated to grow in 3D. Organotypic tumor slices, on the other hand, retain the complexity of tumors *in vivo* without extensive manipulation of the tissue. This leads to a model system in which the tumor cells are surrounded by their original microenvironment, rather than artificial matrices.

The first publications on organotypic tissue slices originate from the 1960s involving cardiac and brain tissue [55,56]. This technique involves precision slicing of tissue using specifically designed machines; the Krumdieck tissue slicer was considered the golden standard, until more recently the vibrating blade microtome (vibratome) was introduced [57]. The Krumdieck tissue slicer punches a cilindrical core from the tissue, which is then sliced by a rotating knife. The vibratome uses a vibrating knife to cut the tissue and has lower mechanical impact. Tissue slicing does not interfere with morphology and functional activity of the tissue and was soon exploited to study many different tissues including liver, retina, prostate, breast and testicular tissue [58–62]. Direct comparison of the Leica VT1200 S vibrating blade microtome and the Krumdieck tissue slicing techniques revealed that the vibratome produces more precise and reproducible slices [61]. However, this may not be true for all tumor types. For example, the Krumdieck tissue slicer outperforms the vibratome when slicing the viscous texture of glioblastomas [63].

*Ex vivo* drug screens and other functional tests require optimal culture conditions for these organotypic tumor tissue slices. Tumor slicing is usually achieved within hours of surgical resection of the primary tumor to minimize deterioration of the tissue and loss of cellular viability [64]. Short-term culture of tumor tissue slices can be achieved without extensive optimization of culture conditions. In some cases, short-term culture of tissue slices suffices for selection of optimal treatment strategies. For example, a functional assay for homologous recombination capacity has been established. This test exploits RAD51 accumulation at DNA double strand breaks after *ex vivo* irradiation of tumor slices or biopsies to select breast cancer patients for targeted treatment with PARP inhibitors [64].

However, preservation of tumor slices for extended periods without losing tumor viability, necessary for *ex vivo* drug screening, required extensive optimization of media composition and/or culture conditions.

Culture conditions can generally be divided in slices cultured on the bottom of the dish, freely floating in the medium or grown on membrane supports. This can be combined with rotational movement of the cultures to achieve optimal diffusion of oxygen and nutrients. Some studies report growth under low oxygen conditions [65], but this in general leads to low tumor slice viability. Culture media that have been used are very diverse. The basis is generally one of the commercially available media for cell culture, supplemented with fetal bovine serum and antibiotics. Furthermore, various growth factors have been added to optimize conditions for specific tumor types.

Tissue slices can be cultured on Teflon membrane inserts, which have 0.4-μm pores that allow preservation of 3D tissue structure in culture and position the tissue slice at the air/liquid interface enabling efficient oxygenation. Colon, lung, head and neck, gastric, esophageal and prostate cancer slices have been reported to be preserved by incubation on Teflon membrane inserts [66–68]. Davies *et al*. have extensively studied the impact of various incubation methods [65]. They found that tumor transportation and slicing had little impact on stress protein expression, whereas different cultivation methods significantly changed tissue vitality and expression of stress proteins. Vitality of tumor slices of various origins was maintained better when cultured on a membrane support compared with on the bottom of a culture dish. Although, even under these conditions, changes were observed in the slices after a few days in culture. Cultivation of the slice on the bottom of a culture dish led to significant alteration of a number of stress pathways and loss of tissue integrity, which can probably be explained by lack of oxygen and nutrient exchange. To overcome this issue, tissue slices can be incubated while floating in medium, which can be achieved via continuous movement using an orbital shaker. Breast cancer slice viability was preserved for prolonged periods of time when slices were incubated under constant rotation. Slices from the same breast tumor cultured under rotation showed more proliferating cells after 48 h compared with slices cultured in static conditions [69]. Breast cancer slices, obtained via vibratome slicing and cultured under constant rotation, remained vital for 7 days [69] (Table 1).

**Table 1.** **Comparison of various reports on organotypic tumor slices.**

**Study (year)**	**Tissue**	**Slicing method**	**Specific culture condition**	**Validation tool for culture conditions/assay read-out**	**Duration of successful culture**	**Investigated compound**	**Study size**	**Ref.**
Van der Kuip *et al*. (2006)	Breast	Krumdieck	- Composite medium – Rotation (150 rpm) – 200 μm slices	– TMRM/SYTO-63/picogreen three-color assay	4 days	– Taxol	22	[72]
Vaira *et al*. (2010)	Colon Lung Prostate	Vibratome	– Culture plate inserts – No rotation – 400 μm slices	– Ki-67 staining – MTT assay – TUNEL assay (apoptosis) – BrdU incorporation – p-Akt and p-S6RP protein levels	5 days	– LY294002 (PI3K inhibitor)	42	[68]
Davies *et al*. (2015)	Breast Prostate Lung	Vibratome	– Composite medium – No rotation – Culture plate inserts – 200–300 μm slices -High/low oxygen	– Ki-67 staining – CICK18 stainig (apoptosis) – Cleaved caspase 3 staining – qPCR and IHC analysis of several other biomarkers – Morphologic examination	4 days	NA	No cohort	[65]
Holliday *et al*. (2013)	Breast	Vibratome	– Regular medium – No rotation – 250 μm slices	– MIB1 staining (proliferation) – M30 staining (apoptosis) – Morphological examination	7 days (no quantification)	– Doxorubicin – Tamoxifen	10	[82]
Naipal *et al*. (2016)	Breast	Vibratome	– Composite medium – Rotation (60 rpm) – 300 μm slices	– EdU incorporation – TUNEL assay (apoptosis) – Morphologic examination	7 days	– FAC (5-FU, doxorubicin, cyclophosphamide)	15	[69]
Gerlach *et al*. 2014	Head and neck	Both vibratome and Krumdieck	– Composite medium – Culture plate inserts – 350 μm slices	– Ki-67 staining – Cleaved caspase 3 staining – Morphologic examination	6 days (no quantification)	– Cisplatin – Cetuximab – Docetaxel	No cohort	[66]
Koerfer *et al*. (2016)	Gastric and esophageal	Krumdieck	– Regular medium – Culture plate inserts – No rotation – 400 μm slices	– Cytokeratin – Ki-67 staining – Cleaved caspase 3 staining – Morphologic examination	6 days	– 5-FU – Cisplatin	8	[67]
Carranza *et al*. (2015)	Breast	Krumdieck	– Composite medium – Rotation (25 rpm) – 250–300 μm slices	– Alamar Blue assay – LDH release – Morphologic examination – KI67 staining	3–4 days	– Paclitaxel – Caffeic acid – Ursolic acid – Rosmarinic acid	9	[62]

Many different methods for cultivation of organotypic tissue slices exist and the optimal system remains to be selected. Various reports on organotypic tumor tissue slices used different methods for slice cultivation. Moreover, assays and quality standards differ between reports, making it difficult to draw conclusions.

Prolonged culture of tumor slices is an absolute requirement for investigation of cytotoxic drug responses. Improved efficiency of drug response prediction is clearly needed, since only 7.5% of the anticancer compounds tested in Phase I clinical trials eventually obtains approval [70]. One of the main reasons for this disappointing percentage is the use of preclinical models that do not represent the complexity of *in vivo* tumors [71]. Organotypic tissue slices could serve as a model to examine response of the tumor to anticancer compounds *ex vivo*, as it most closely resembles the heterogeneity and microenvironment of *in vivo* tumors. Indeed, cytotoxic responses to targeted therapies as well as classic chemotherapeutic agents have been predicted in organotypic tissue slices [62,66–69,72]. Also in this case, concordance between *ex vivo* sensitivity and *in vivo* treatment response rates still remains to be validated. For this purpose, pretreatment biopsies should be obtained for *ex vivo* sensitivity assays, subsequently comparing these results with *in vivo* post-treatment response evaluations. Therefore, the tissue slicing technique and incubation should be optimized for biopsy specimens, taking the first steps toward clinical validation and subsequent diagnostic application of this model system.

A major disadvantage of tumor tissue slices as a method for drug testing is its relatively low throughput. The technique is rather laborious and requires specialized analysis tools that may not be easily implemented outside research settings. Markers that are generally used for determining response are analyzed by immunofluorescent microscopy and quantification of these markers is still challenging. Therefore, it is to be expected that this culture system will only be used in a laboratory setting and connected to clinical studies in the near future. Depending on the concordance between *ex vivo* outcomes and tumor response in patients, these methods could be adapted for a more routine clinical setting. However, automation of the processing and read-out is not easily possible and will require technical adaptations such as a cancer-on-chip approach described below.

Hypoxia is another potential problem of organotypic tissue slice cultures as a model system [73]. Because intact vascularity is absent in tissue slices, the amount of oxygen available is limited to gas diffusion. Several parameters influence this oxygen diffusion, such as slice thickness, matrix stiffness, cellularity and metabolic and proliferative activity of the tumor and stromal cells [69,73]. Especially long-term cultures with extensive proliferation of tumor cells may cause hypoxia in the center of these growing tumor slices. On the other hand, organotypic tissue slices may allow detailed investigation of gradients of oxygen tension observed in patient tumors in a controlled setting *in vitro* [73].

A drawback of many model systems, including organotypic tissue slices, is the lack of systemic features such as an immune system. The engineering of personalized tumor ecosystems, which conserve the microenvironment through cultivation of tissue slices in defined tumor grade-matched matrix support and in the presence of autologous serum, may be a next step in organotypic tissue slice cultivation [74]. In these personalized tumor ecosystems, patient serum derived immune cells could infiltrate the tissue slice, extending the possible applications of this model system.

To conclude, organotypic tissue slices represent a solid model system for functional assays and drug sensitivity testing for personalized medicine, due to its fast generation time and reflection of intratumor heterogeneity and tumor–stromal interactions. However, many different methods for cultivation of organotypic tissue slices exist and the optimal system remains to be selected.

Although many publications on tumor slice cultures lack careful comparison of culture conditions and are not easily comparable to each other, a common denominator begins to emerge from the literature. Tissue slices from various tumor types, including lung, prostate, colon, gastric and head and neck cancer have been cultured for several days [66–69]. Glioblastoma tissue slices remained vital and still harbored histological characteristics of the original tumor even after 16 days of culture [63]. Different tumors require different culture conditions. Highly proliferative tumors, for instance, require more oxygen exchange, whereas very fragile tissue slices benefit from incubation on supportive material. Furthermore, each tumor type has its own nutrient and growth factor requirements. For example, several reports on breast cancer tissue slices used addition of insulin [62,69,72].

It is not easy to evaluate the merits of each tumor tissue slice culture system, as different assays and quality standards have been used to characterize tissue quality at various time points (Table 1). Most investigators report on tissue morphology and cell death, although careful quantification is sometimes lacking. However, proliferation is not always monitored over time or different methods were used to assess proliferation. Often proliferation rate is estimated using Ki67 staining and several tissue slicing publications use this same marker. However, this may not faithfully reflect the proliferative state at the time of assay, as Ki-67 is expressed in all phases of the cell cycle, except G0 [75]. Therefore, proliferation should be evaluated with markers for S/G2 phase cells (geminin or cyclin A) or DNA synthesis (EdU incorporation), which measure active proliferation directly.

We propose a minimal standard, which should be performed for each tissue slice culture method, to enhance transparency and improve comparison between experiments and research groups. This standard should at least include morphology, proliferation and apoptosis of the tumor cells assessed up to 7 days of incubation. Moreover, it is of utmost importance to report all culture conditions used, instead of only those achieving optimal results. This should allow selection of the optimal culture system for organotypic tissue slices which can subsequently be adopted as the standard in the field of personalized medicine and drug testing.

## Cancer-on-chip

New 3D culture systems incorporate advances in biomaterials, microfluidics and tissue engineering to improve culture quality and reproducibility. Cancer-on-chip is a general term to describe various 3D microculture systems to maintain tumor cells in a controllable microenvironment. For example, cultivation of difficult-to-preserve primary patient-derived multiple myeloma cells has been achieved in a device consisting of a 3D tissue scaffold constructed in a perfused microfluidic environment [76]. Recent progress in the cancer-on-chip field, specifically in hydrogel-incorporated microfluidics for long-term cell maintenance and exploitation of these culture devices for automated bioassay applications was reviewed by Lee *et al*. [77]. Specific microfluidics devices have been designed to study metastasis formation as well as personalized immunotherapy [78,79].

Up to date, most cancer-on-chip systems facilitate cultivation of tumor cells. Yet, organotypic tissue slices can be inserted into these microfluidic devices as well, enabling long-term culturing with decreased handling of tissue slices. The conditions in these devices can be very similar to *in vivo* conditions, with constant supply of nutrients, waste removal and controlled access to oxygen. Moreover, endothelial barriers and interstitial pressure can also be mimicked in the more elaborate versions of these cancer-on-chip set-ups [80]. Thereby, the maximum time that slices remain vital in culture could be expanded and cultivation will be more high-throughput compared with original organotypic tissue slice cultures [81]. Optimization of the exact geometry and growth conditions of these microfluidics set-ups hold great promise for tumor slice culturing and development of predictive diagnostic assays. Although 3D microculture systems have been developed, this technique requires extensive optimization to achieve systems facilitating tissue slice cultivation.

## Conclusion & future perspective

Patient stratification is of utmost importance in the era of personalized medicine. Selection of patients for precision therapies should ideally be based on the tumor phenotype. Functional *ex vivo* assays may be the ultimate selection method when unique molecular markers have not been identified for particular drugs.

**Figure 2.**
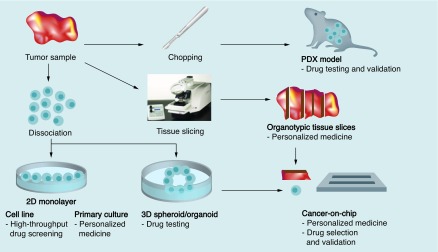
**Main applications of different *ex vivo* model systems.** An *ex vivo* model system should be chosen according to the purpose of the specific research. Each *ex vivo* model system has its own benefits and disadvantages, making one more applicable for a specific research endeavor than the others.

Approaches for patient stratification should be fast, simple and widely applicable to many tumor types or subtypes without being biased for cell selection and tumor heterogeneity. As generation time of organotypic tissue slices is very fast and results can be obtained within days, this model is in principle suitable for drug selection in the personalized medicine era, whereas 2D monolayers, 3D organoids and PDX models require longer generation times. On the other hand, organoids and 2D monolayers can be exploited for high-throughput drug screenings, yet tissue slices remain a low-throughput technique. This indicates that selecting the right model system for the right purpose is at least as important as developing new and improved culture systems (Figure 2). Therefore, a thorough understanding of the advantages and drawbacks of each culture method is important.

In the future, developments in the field of cancer-on-chip might integrate the best of both worlds, incorporating tumor heterogeneity and tumor–stroma interactions represented in organotypic tissue slices in a more high-throughput fashion.

**Executive summary**

- Selecting the right model system for the right purpose is at least as important as developing new and improved culture systems.
- High-throughput drug screening requires 2D or 3D tumor cell cultures, whereas patient-derived xenograft models are useful for validation purposes.
- Organotypic tissue slices reflect intratumor heterogeneity and tumor–stromal interactions of *in vivo* tumors.
- Functional assays on organotypic tissue slices can be evaluated in a few days to weeks, making them suitable for drug selection in the personalized medicine era.
